# Construction and validation of a predictive model for post-operative kinesiophobia in total hip arthroplasty patients

**DOI:** 10.3389/fmed.2025.1699157

**Published:** 2025-11-24

**Authors:** Sunjuan Dong, Xiucheng Guo, Yanling Zhou, Yinzhong Chen, Chi Wang, SiJing Peng, Li Wu, Wenxi Han, Weiting Liu

**Affiliations:** 1 School of Nursing, Anhui University of Chinese Medicine, Hefei, Anhui, China; 2 The Second Affiliated Hospital of Shandong First Medical University, Tai’an, Shandong, China; 3 Key Laboratory of Geriatric Nursing and Health, Anhui University of Chinese Medicine, Hefei, Anhui, China

**Keywords:** total hip arthroplasty, kinesiophobia, influencing factors, risk prediction mode, nomogram

## Abstract

**Background:**

Total hip arthroplasty (THA) is an effective treatment for end-stage hip disease, which can significantly relieve pain, rebuild joint function, and improve patients’ quality of life. Early post-operative functional exercises are essential to promote the recovery of hip function and minimize complications. However, some patients are afraid of exercise due to the risk of pain or dislocation of the prosthesis, resulting in poor adherence to rehabilitation exercises. Kinesiophobia has now become a key factor hindering the rehabilitation process and leading to poor functional recovery. Therefore, early identification of high-risk groups and implementation of targeted interventions are essential to improve patient prognosis.

**Objectives:**

To construct and validate a risk prediction model for post-operative kinesiophobia in total hip arthroplasty (THA) patients.

**Methods:**

This study is a single-center cross-sectional study with only internal validation. Through the convenience sampling method, 205 patients who underwent total hip arthroplasty (THA) at the Second Affiliated Hospital of Shandong First Medical University from October 2024 to May 2025 were selected as the study subjects. Independent predictors associated with the occurrence of kinesiophobia were screened by univariate analysis and multifactorial logistic regression analysis. A nomogram risk prediction model was constructed using R software, and the Hosmer-Lemeshow test, receiver operating characteristic (ROC) curve, area under the curve (AUC), calibration curves, and decision analysis curves (DCA) were used to evaluate the model’s goodness-of-fit, discriminability, calibration, and clinical utility, respectively. The Bootstrap method was used to perform 1,000 repeated samplings for internal validation of the model.

**Results:**

A total of 126 out of 205 post-operative THA patients developed kinesiophobia, and the incidence of kinesiophobia was 61.5%. Logistic regression showed that pain history duration, anxiety, hip function, coping style, and rehabilitation self-efficacy were the influencing factors for the development of kinesiophobia in post-operative patients with THA (*P* < 0.05). The AUC value of the constructed model was 0.947 (95% CI 0.919∼0.975), the specificity was 0.873, and the sensitivity was 0.911. The Hosmer-Lemeshow test (χ*^2^* = 2.287, *P* = 0.971) and the calibration curve showed that the predicted probability of the model was consistent with the probability of the actual occurrence, and the model predicted the effect well; the DCA curve indicates that the model has good clinical utility. Internal validation using the Bootstrap method yielded an AUC value of 0.912 (95% CI 0.875–0.949).

**Conclusion:**

THA patients with a pain history duration (> 1 year), anxiety, poor hip function, low active coping, and poor rehabilitation self-efficacy are more likely to develop kinesiophobia after surgery. The constructed model has good predictive efficacy, which is helpful for early clinical identification of high-risk patients and provides a reference for developing individualized interventions.

## 1 Introduction

Total hip arthroplasty (THA) is the replacement of diseased or dysfunctional hip joints with artificial prosthesis, which can effectively alleviate pain, rebuild joint function, and improve the quality of life of patients, and it is currently the most effective treatment for end-stage hip joint diseases (1, 2). With the aging of the population and the advancement of medical technology, the number of THA surgeries continues to climb, and according to statistics, about 2 million patients worldwide undergo hip replacement surgery every year (3). The key to the success of surgery lies not only in the precise implantation of the prosthesis, but also in whether the patient can maximize the recovery of joint function after surgery. Studies have shown that (4) the degree of recovery of hip joint function after surgery is closely related to the rehabilitation exercise of patients after surgery, and early functional exercise can promote the recovery of hip joint function, shorten the hospitalization time, and reduce the occurrence of post-operative complications. However, some patients developed kinesiophobia after surgery, resulting in poor compliance with rehabilitation exercises.

Kinesiophobia is a common psychological problem in post-operative patients with THA, which refers to excessive and irrational fear of physical movement or physical activity due to pain or fear of secondary injury (5). It has been found (6) that kinesiophobia is a key factor affecting the prognosis of THA patients. This fear is not simply pain avoidance, but rather a misperception developed by the patient that activity will inevitably lead to loosening of the prosthesis, dislocation, increased pain, or even catastrophic consequences (7). This misperception will cause patients to develop a “fear-avoidance” behavior: even if they have the ability to move, they will actively limit the range of activities, reduce the intensity of rehabilitation training, prolong the bedtime, and even refuse the necessary early functional exercises due to fear, which will ultimately slow down the process of rehabilitation and affect the effect of joint recovery (8). Therefore, early prediction of the risk of post-operative kinesiophobia in THA patients is crucial for improving patients’ compliance with rehabilitation exercises and improving prognosis.

In recent years, kinesiophobia has gradually gained attention in the field of clinical research. However, there are obvious limitations in current studies, most of which focus on the investigation of the current status of kinesiophobia in THA patients and the analysis of its influencing factors. Moreover, the lack of in-depth understanding of the occurrence of kinesiophobia in THA patients after surgery has led to limited clinical assessment capabilities and difficulties in carrying out effective interventions. Therefore, it is necessary to improve the ability to recognize high-risk groups. In existing studies, there is a relative paucity of content related to risk prediction models for post-operative kinesiophobia in THA patients, and there is a lack of effective and convenient tools to predict the risk of post-operative kinesiophobia in THA patients. The nomogram risk prediction model has significant advantages as a commonly used risk assessment tool in clinical practice. It is able to achieve individualized and visualized prediction of the risk of adverse events by assigning values to risk factors and transforming them into graphical form. The model has been successfully applied to individualized kinesiophobia occurrence risk prediction in patients with lung cancer (9), lumbar disk herniation (10), and after coronary intervention (11). Therefore, in this study, we analyzed the influencing factors of post-operative kinesiophobia in THA patients with whether or not kinesiophobia occurs in THA patients as the dependent variable, constructed a nomogram risk prediction model, which is conducive to the early identification of high-risk patients by healthcare professionals, and provided a reference for the development of targeted intervention strategies to break the vicious circle of “fear-braking-functional deterioration,” and improve the long-term prognosis of patients.

Previous studies have shown that the degree and duration of pain, anxiety, and depression status, level of social support, self-efficacy, coping styles, and hip function are all closely related to the occurrence of kinesiophobia after THA (12–16). Among these factors, pain affects patients’ psychological state and willingness to recover, and when the degree of pain experienced by patients is heavier and lasts longer, the motor fear generated within them tends to be more intense. This is due to the fact that pain causes patients to subconsciously associate exercise with pain, thus instinctively resisting exercise and further hindering the recovery process (17). Whereas anxiety and depression will enhance the stress response of the patient’s body and affect the coordination and flexibility of the muscles around the hip joint, when the patient performs rehabilitation exercises, more discomfort will be generated due to the limited muscle function. This discomfort will further aggravate the patients’ psychological burden, causing them to worry about the effect of the surgery and lack confidence in the recovery of their own body functions, which in turn reduces their acceptance of exercise (18). In addition, a strong social support network consisting of family members, friends, and healthcare professionals can effectively promote functional recovery, emotional stability, and the ability to live independently in the long term; conversely, a lack of support may impede the recovery process and lead to poor post-operative outcomes (19). At the same time, demographic information such as age, gender, education level, and healthcare payment method is similarly associated with the development of kinesiophobia (20). When identifying patients with possible kinesiophobia after THA, all of these types of factors should be considered in order to more fully and accurately assess the patient’s condition.

The biopsychosocial model (BPS) is a model proposed by Engel for describing the behavioral and psychological processes of individuals, emphasizing the interrelationships between mind, body, physiology, and psychology, which suggests that an individual’s physical and mental health is closely related to biological factors (e.g., personal background, current status of the disease), psychological factors (e.g., emotion, cognition), and social factors (social support, economic status, etc.) (21). Based on the theoretical framework of the bio-psycho-social model, this study selected research variables from four dimensions, namely, physiology and disease (pain level and duration, hip function), cognition and beliefs (rehabilitation self-efficacy, coping styles), emotion (anxiety, depression), and society and environment (social support, demographic and sociological factors), to explore the risk factors for the development of kinesiophobia in post-operative patients with THA and to construct a nomogram prediction model, through which high-risk patients can be identified early and intervened in a timely manner, so as to reduce the incidence of post-operative kinesiophobia in THA, help patients to recover joint function early and improve their quality of life.

## 2 Materials and methods

### 2.1 Study design and target population

This is a single-center cross-sectional study, which facilitated the selection of 205 patients who underwent THA treatment in the Second Affiliated Hospital of Shandong First Medical University from October 2024 to May 2025, and the patients were categorized into kinesiophobia and non-kinesiophobia groups according to whether or not they developed kinesiophobia after surgery. Inclusion criteria: first-time THA surgery; age ≥ 18 years old; clear consciousness, expression ability is OK; voluntary participation in this study, and signed the informed consent. Exclusion criteria: patients with psychiatric history or cognitive impairment; patients with other important organ injuries, whose conditions may change at any time; patients with other diseases that limit lower limb activities.

In this study, the sample size was calculated using the Events Per Variable (EPV) method (22), and 18 study variables were finally selected in this study after combining the literature review, clinical practice experience, and group discussion, with an EPV ≥ 5 set to ensure the stability of the results. Based on the prevalence of kinesiophobia after total hip arthroplasty (THA) in a previous study (23), which was 58.7%, and taking into account the 20% loss to follow-up rate, the sample size was calculated to be at least (18 × 5/0.587)/0.8 = 192 cases. 205 valid questionnaires were finally recovered in this study.

### 2.2 Data collection

In this study, two uniformly trained researchers worked together to collect data and initially screened eligible THA patients through the electronic medical record system based on pre-established inclusion and exclusion criteria. Before the formal data collection, the researchers will fully communicate with the patients and their families, informing them in detail about the purpose, content, and process of the study, and promising to strictly protect the privacy of the patients and never disclose any personal information, and signing the informed consent form after obtaining the understanding of the patients and their families. General information was extracted directly through the hospital information technology platform, and other information was collected using a paper version of the scale when the patients’ post-operative vital signs were stable and their mental status was good. Before filling in the scale, the researchers used a unified instruction to explain the requirements and precautions for filling in the scale. For patients with dyslexia or those who were unable to complete the scale independently for other reasons, the researchers would adopt an objective and neutral way of expression, explaining the content of the scale one by one and avoiding inducing questions, and assisting the patients in completing the scale in the form of questions and answers. After the completion of the questionnaire, the researchers collected the questionnaires on the spot, evaluated and reviewed the questionnaires, and excluded the questionnaires with obvious errors. All data were entered independently by two researchers and double-checked to ensure the accuracy of the final data.

### 2.3 Measures

#### 2.3.1 General information questionnaire

Self-designed questionnaire through literature review and group discussion, where demographic information included gender, age, place of residence, marital status, monthly household income, literacy level, and healthcare payment method. Disease information included BMI, pain level (visual analog scale), years of pain, and presence of comorbid chronic diseases.

#### 2.3.2 Tampa scale for kinesiophobia

The Tampa Scale for Kinesiophobia was developed by Miller et al. (24) and Chineseized by Hu (25), using a four-point Likert scale, with scores of 1–4 indicating “completely oppose, oppose, agree, and completely agree,” and a total of 17 entries, of which four entries (entries 4, 8, 12, and 16) were reverse scored. There were 17 entries, four of which (entries 4, 8, 12, 16) were reverse scored, with total scores ranging from 17 to 68, and >37 points were recognized as kinesiophobia. The Cronbach’s alpha coefficient of this scale was 0.894 in this study.

#### 2.3.3 Simplified coping style questionnaire

The Simplified Coping Style Questionnaire was compiled by Xie (26), using a four-point Likert scale, with scores of 0–3 as “do not take, occasionally take, sometimes take, and often take,” respectively. A total of 20 entries were divided into two dimensions: positive coping (entries 1–12) and negative coping (entries 13–20), and the dimension with the higher score was the coping style that the patients preferred to adopt. The Cronbach’s alpha coefficients of the positive coping and negative coping dimensions of this scale were 0.880 and 0.858, respectively, in this study.

#### 2.3.4 Social support rating scale

The Social Support Rating Scale was developed by Xiao (27), with a total of 10 entries, including three dimensions of subjective support (entries 1, 3, 4, and 5), objective support (entries 2, 6, and 7), and utilization of support (entries 8, 9, and 10), and a four-point Likert scale was used, i.e., 1 point was assigned to option (1), and four points were assigned to option (4), where 6 and 7 were assigned 0 points on a scale of 0–9, and 0 points were assigned to option 7, which was assigned “no source.” Four points, of which questions 6 and 7 were scored on a scale of 0–9, with A scored 0, I scored 9, and question 7 scored 0 for “no source.” A total score of <20 means low social support, a total score between 20 and 30 means some social support, and a score of 30 or more means satisfactory social support. The Cronbach’s alpha coefficient of this scale was 0.893 in this study.

#### 2.3.5 Hospital anxiety and depression scale

The hospital anxiety and depression scale was compiled by Zigmond et al. (28) and consists of 14 entries, including two subscales of anxiety and depression, where the odd-numbered entries are for anxiety and the even-numbered entries are for depression, and a Likert four-point scale was used, with each entry scored from 0 to 3, and a dimensional score of >7 indicating that the patient suffers from anxiety or depression. The Cronbach’s coefficients of the anxiety and depression sub-scales were 0.869 and 0.870, respectively, in this study.

#### 2.3.6 Self-Efficacy for rehabilitation outcome scale

The Self-Efficacy for Rehabilitation Outcome Scale was developed by Waldrop et al. (29) and Chineseized by Wang et al. (30), with a total of 12 entries and a Likert-11 scale, with scores ranging from 0 to 10, which indicate “cannot at all” to “do not have any difficulty,” respectively. A total of 12 entries were scored on a Likert-11 scale from 0 to 10, indicating “no difficulty at all” and “no difficulty at all,” respectively, with a total score of 120 points, and the higher the score, the more confident the patient is in rehabilitation training. The Cronbach’s alpha coefficient of this scale was 0.907 in this study.

#### 2.3.7 Harris Hip Scoreing System

Harris Hip Scoreing System was compiled by Harris (31), the higher the score indicates that the patient’s hip function is better, the scale mainly includes four aspects of pain (44 points), function (47 points), deformity (four points), mobility (five points), the total score of 100 points, of which 90–100 points is excellent, 80–89 points is good, 70–79 was classified as fair, and ≤ 69 was classified as poor. The Cronbach’s alpha coefficients for this scale ranged from 0.830 to 0.910.

### 2.4 Statistical analysis

Data were statistically analyzed using SPSS 29.0 and R4.4.2 software. Normally distributed quantitative data were expressed as mean ± SD, and qualitative data were expressed as frequency and percentage (%); comparisons between groups were made using two independent samples *t*-test and chi-square test. Quantitative data that did not conform to the normal distribution were expressed as median and quartiles [M(P25, P75)], and the Mann-Whitney rank-sum - sum test was used for comparison between groups. Qualitative data were described by the number of cases and percentage (%), and the χ^2^-test, Fisher’s exact probability method, and rank-sum test were used for comparison between groups. Variables that were statistically significant in the one-way analysis were included in the multifactor Logistic regression analysis, and the stepwise forward-backward method was used to screen the independent predictors, while the multicollinearity between the predictor variables was checked by the Variance Inflation Factor (VIF), which indicated the existence of multicollinearity between the variables when the VIF was ≥5; finally, the predictive model was constructed using the R software, and the nomogram was drawn. The predictive ability of the model was evaluated using the Hosmer-Lemeshow goodness-of-fit test and the Area Under the Curve (AUC) of the receiver operating characteristic (ROC) curve, and the AUC value was close to 1, which indicated that the model had good predictive ability. The calibration and clinical utility value of the model were evaluated by the calibration curve and Decision Curve Analysis (DCA). And the model was also internally validated by repeated sampling 1,000 times using the Bootstrap method with a sample size of 205 cases per sampling to assess the stability of the model. The difference was considered statistically significant at two-sided *P* < 0.05.

### 2.5 Ethical considerations

The study followed the Declaration of Helsinki and was approved by the Ethics Committee of the Second Affiliated Hospital of Shandong First Medical University (2024-H-053).

## 3 Results

### 3.1 Current status of post-operative kinesiophobia in THA patients

The 205 THA patients were categorized into kinesiophobic and non-kinesiophobic groups according to whether or not they developed kinesiophobia postoperatively, of which 126 developed kinesiophobia postoperatively, with a kinesiophobia incidence rate of 61.5%.

### 3.2 Univariate analysis of factors influencing post-operative kinesiophobia in THA patients

The results of the univariate analysis of the two groups of patients showed statistically significant differences in gender, age, place of residence, monthly household income, presence of comorbid chronic diseases, pain history duration, anxiety, depression, social support, coping styles, rehabilitation self-efficacy, and hip function (*P* < 0.05), as shown in Table 1.

**TABLE 1:** Univariate analysis of post-operative development of kinesiophobia in total hip arthroplasty patients.

Variables	Non-kinesiophobic group (*N* = 79)	Kinesiophobia group (*N* = 126)	Statistics	*P*-value
Gender (%)			23.380^1^	<0.001
Male	51 (64.60)	38 (30.20)	–	–
Female	28 (35.40)	88 (69.80)	–	–
Current address (%)			13.240^1^	<0.001
Urban	39 (49.40)	31 (24.60)	–	–
Rural	40 (50.60)	95 (75.40)	–	–
Educational level (%)			0.307^1^	0.989
Primary school and below	21 (26.60)	31 (24.60)	–	–
Middle school	33 (41.80)	52 (41.30)	–	–
High school/technical secondary school	12 (15.20)	19 (15.10)	–	–
Junior college	7 (8.90)	12 (9.50)	–	–
University and above	6 (7.60)	12 (9.50)	–	–
Marital status (%)			0.738^1^	0.691
Widowed	0 (0.00)	1 (0.80)	–	–
Unmarried	1 (1.30)	1 (0.80)	–	–
Married	78 (98.70)	124 (98.40)	–	–
Medical payment methods (%)			5.560^1^	0.062
Urban and rural residents’ medical insurance	47 (59.50)	72 (57.10)	–	–
Urban workers’ medical insurance	17 (21.50)	15 (11.90)	–	–
Out-of-pocket expenses	15 (19.00)	39 (31.00)	–	–
Monthly household income (%)			59.395^1^	<0.001
<3,000	9 (11.40)	76 (60.30)	–	–
3,000–5,000	44 (55.70)	45 (35.70)	–	–
≥5,000	26 (32.90)	5 (4.00)	–	–
Presence of comorbid chronic diseases (%)			33.201^1^	<0.001
No	58 (73.40)	39 (31.00)	–	–
Yes	21 (26.60)	87 (69.00)	–	–
Number of years of pain (%)			168.216^1^	<0.001
>1	14 (17.70)	97 (77.00)	–	–
≤1	65 (82.30)	29 (23.00)	–	–
Age	58.85 ± 11.66	68.44 ± 11.61	−5.746^2^	<0.001
BMI	24.71 ± 3.67	24.61 ± 3.84	0.201^2^	0.841
Pain numeric score	1.42 ± 1.64	1.59 ± 1.63	−0.725^2^	0.469
Anxiety	2.86 ± 1.91	8.91 ± 3.81	13.120^2^	<0.001
Depression	3.95 ± 4.27	8.37 ± 4.50	−6.970^2^	<0.001
Social support score	51.38 ± 5.33	36.16 ± 8.13	14.760^2^	<0.001
Rehabilitation self-efficacy score	99.52 ± 4.93	75.10 ± 14.72	14.240^2^	<0.001
Active coping style score	29.73 ± 2.87	16.50 ± 6.30	17.550^2^	<0.001
Passive coping style score	7.65 ± 2.50	13.93 ± 4.70	−10.930^2^	<0.001
Harris hip function score	66.29 ± 9.29	43.89 ± 11.91	14.220^2^	<0.001

^1^Is the χ2-value. ^2^Is the *t*-value.

### 3.3 Logistic regression analysis of factors influencing post-operative kinesiophobia in THA patients

The occurrence of kinesiophobia was used as the dependent variable (yes = 1, no = 0), and the variables that were statistically significant in the univariate analysis were used as the independent variables. Logistic regression (stepwise forward-backward method) was chosen to screen for independent predictors, and a table of the independent variable assignments is shown in Table 2. Variance Inflation Factor (VIF) tests were performed, and all variables had VIF values < 2, indicating no covariance and good model fit. The results showed that pain history duration, anxiety, positive coping styles, rehabilitation self-efficacy, and Harris hip function were the most important predictors of the occurrence of THA in patients with predictors of kinesiophobia, see Table 3.

**TABLE 2:** Assignment of independent variables.

Variables	Assignment of values
Gender	Male = 0, female = 1
Current address	Urban = 0, rural = 1
Monthly household income	<3,000 = 0, 3,000–5,000 = 1, ≥5,000 = 2
Presence of comorbid chronic diseases	No = 0, yes = 1
Pain history duration	>1 = 0, ≤1 = 1
Age	Substituting the original value into
Anxiety	Substituting the original value into
Depression	Substituting the original value into
Social support score	Substituting the original value into
Rehabilitation self-efficacy score	Substituting the original value into
Coping style score	Substituting the original value into
Harris hip function score	Substituting the original value into

**TABLE 3:** Logistic regression of factors influencing the development of post-operative kinesiophobia in total hip arthroplasty (THA) patients.

Variables	β	SE	Wald χ*^2^*	OR (95% CI)	*P*
Constant	56.311	24.91	5.11	–	0.024
Pain history duration^1^	−5.426	2.507	5.147	0.004 (0.000–0.599)	0.030
Anxiety	1.928	0.849	5.822	6.876 (1.302–36.308)	0.023
Rehabilitation self-efficacy	−0.331	0.168	3.483	0.718 (0.517–0.998)	0.049
Active coping style	−1.159	0.450	6.444	0.314 (0.130–0.758)	0.010
Harris hip function	−0.204	0.097	3.787	0.815 (0.674–0.986)	0.036

^1^The reference for the pain history duration is >1 year.

### 3.4 Construction of a nomogram prediction model for post-operative kinesiophobia in THA patients

**FIGURE 1:**
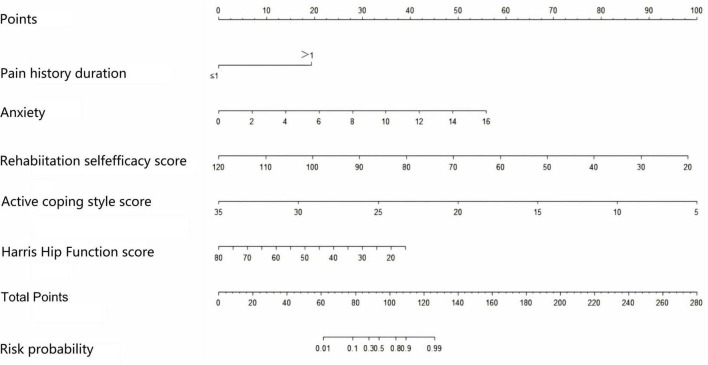
Nomogram risk prediction model for fear of movement after total hip arthroplasty (THA) surgery.

According to the logistic regression results of the post-operative kinesiophobia prediction model for THA patients plotted with the R software, each predictor is located on the left side of the Nomogram, and the corresponding scores are obtained by making a vertical line upward from them, and these scores are added to obtain the total score, and the predicted probability of the patient’s development of kinesiophobia in the post-operative period can be obtained by making a vertical line downward from the coordinate axis of the total score (Figure 1). For example, a patient who underwent THA surgery with a pain history duration of more than 1 year has an anxiety score of 12, a rehabilitation self-efficacy score of 50, an active coping score of 10, and a hip function score of 30. In the Nomogram, each variable has a corresponding score. Thus, pain history duration of more than 1 year corresponds to a score of 19, anxiety status corresponds to a score of 42, rehabilitation self-efficacy corresponds to a score of 69, active coping corresponds to a score of 82, and hip function corresponds to a score of 30. The predicted probability of kinesiophobia in a post-operative patient with THA was obtained by summing the scores for each variable to obtain a total score of 242, and then drawing a vertical line at the total score. Thus, the Nomogram model predicted the probability of kinesiophobia in this patient after surgery was 85%.

### 3.5 Evaluation and validation of a nomogram prediction model for post-operative kinesiophobia in THA patients

**FIGURE 2:**
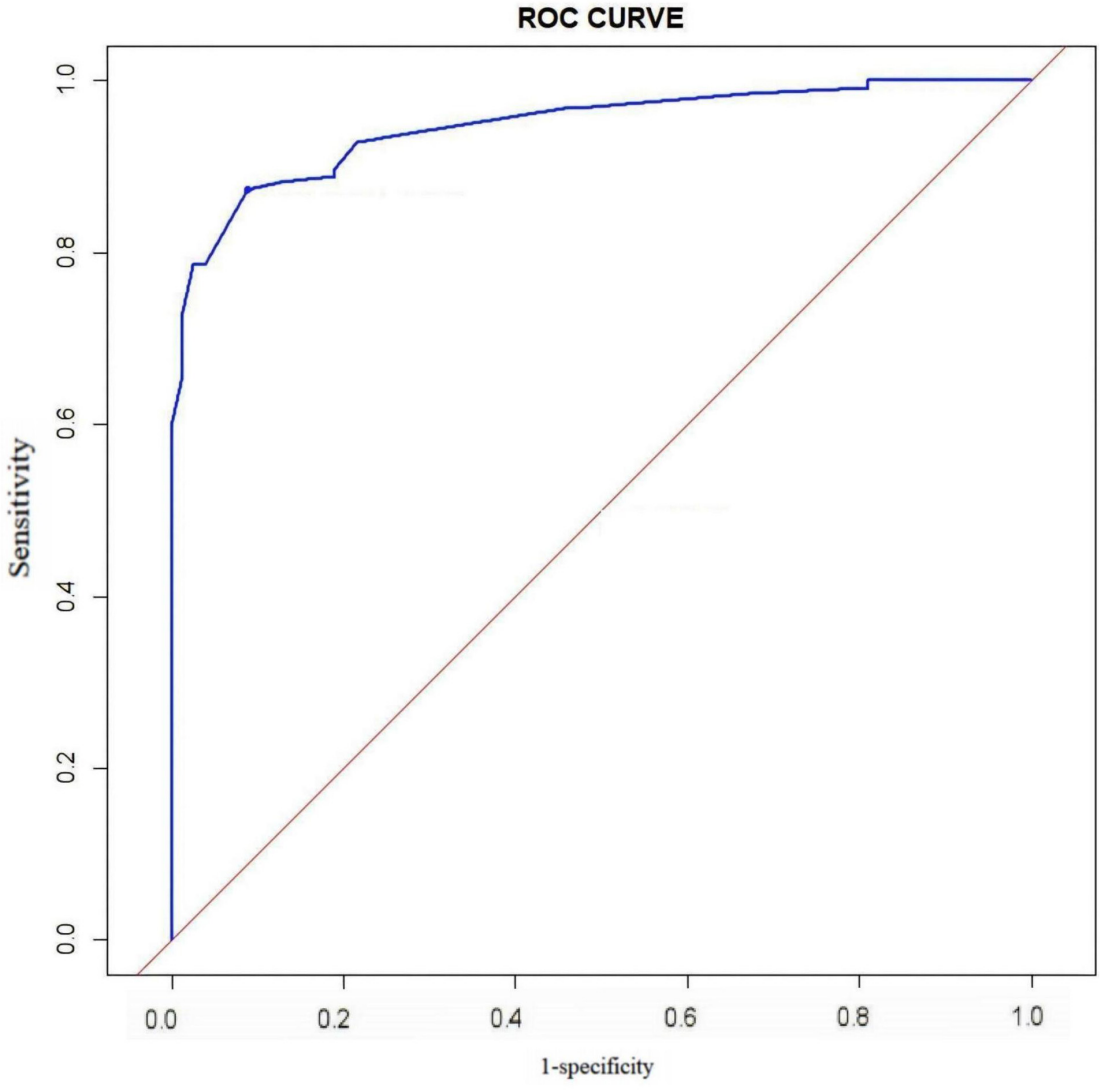
Receiver operating characteristic (ROC) curve of the predictive model for the risk.

**FIGURE 3:**
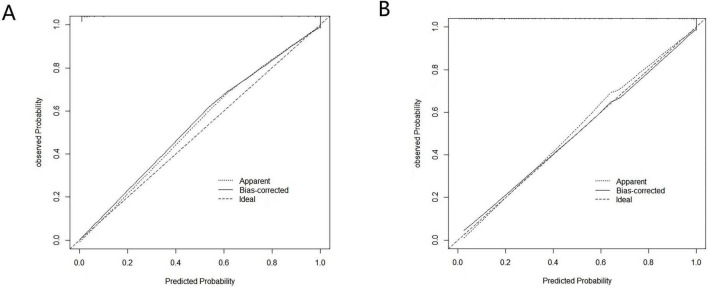
Calibration curve of the predictive model. **(A)** Calibration curve of the original model. **(B)** Calibration curve after 1,000 bootstrap resamples for internal validation.

The ROC curve of this study (Figure 2) shows that the AUC value of the model is 0.947 (95% CI 0.919∼0.975), the specificity is 0.873, the sensitivity is 0.911, and the Hosmer-Lemeshow test χ^2^ = 2.287, *P* = 0.971, which suggests that the model has a better differentiation, fit, and prediction ability. The horizontal coordinate of the calibration curve is the probability of the event predicted by the model, and the vertical coordinate is the probability of the event actually observed. When the calibration curve is closer to the 45° diagonal, it indicates that the predicted probability is closer to the actual probability. The calibration curve in this study is close to the ideal curve (Figure 3A), indicating that there is consistency between the predicted and actual values.

The model was internally validated using the Bootstrap method with 1,000 repeated samples. The result yielded an AUC value of 0.912 (95% CI 0.875–0.949), and the calibration curve fitted well with the ideal curve (Figure 3B), indicating that the model had a good predictive effect.

**FIGURE 4:**
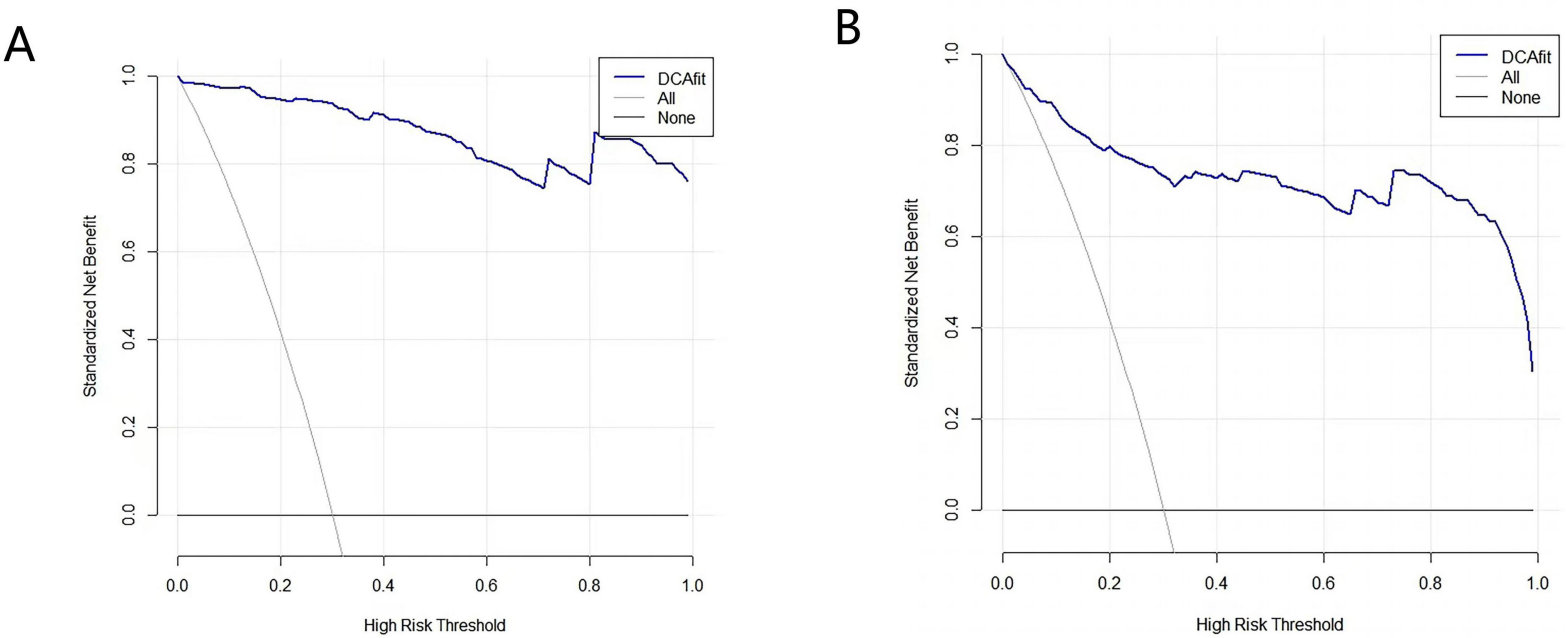
Clinical decision curve of the predictive model. **(A)** Decision analysis curves (DCA) of the original model. **(B)** DCA after 1,000 bootstrap resamples for internal validation.

The decision analysis curve (DCA) is a tool for assessing the utility and clinical application value of clinical predictive models by plotting decision curves to compare the clinical benefits of different predictive models at different therapeutic decision thresholds. The horizontal axis of the curve represents the threshold probability, and the vertical axis represents the net benefit. The solid black line refers to no therapeutic intervention for any patient, the dashed black line refers to therapeutic intervention for all patients, and the blue curve refers to the overall net benefit of that predictive model over the entire range of thresholds. In the threshold range at the bottom of the horizontal axis, the net gain of the predictive model is higher than both the ALL line and the None line, indicating that the model has practical value; when the blue curve is very close to or coincides with the two extreme lines, the ALL line and the None line, it indicates that the model may be of less value for application. The blue curves in this study were between the thresholds 0–1, and the curves were all above the reference line, indicating that the model has a high net benefit and good clinical utility (Figure 4).

## 4 Discussion

### 4.1 Current status of the occurrence of post-operative kinesiophobia in THA patients

The results of this study showed that the incidence of post-operative kinesiophobia in THA patients was 61.5%, which was similar to the findings of Huang Li et al. (23) (58.7%) and higher than the findings of Lai et al. (32) (43.78%), and the reasons for this analysis may be due to the fact that the age of the population included in the present study is on the high side, which may affect the patients’ psychological tolerance and the degree of their fear of post-operative rehabilitation and exercise; and secondly, there are differences in the various studies differed in the time of assessment of kinesiophobia, and the physical recovery and psychological state of patients differed at different post-operative time nodes, which may lead to fluctuations in the assessment of the incidence of kinesiophobia; in addition, the geographical environment is an important factor. There are differences in medical conditions, cultural backgrounds, and patients’ cognitive levels of diseases in different regions, which may have an impact on the occurrence of post-operative exercise phobia among patients. Kinesiophobia, as a kind of excessive fear of pain or injury, will cause patients to avoid necessary activities and functional exercises, which will hinder the recovery of joint function and delay the post-operative rehabilitation process (33). And some studies have shown (34) that the effects of kinesiophobia on patients may last for several years or even accompany patients throughout their lives. Therefore, early identification and intervention of kinesiophobia is essential to improve the prognosis of patients.

### 4.2 Analysis of factors influencing the occurrence of kinesiophobia in THA patients after surgery

#### 4.2.1 Pain history duration

This study showed that a pain history of more than 1 year was a risk factor for the occurrence of kinesiophobia in THA patients after surgery, which was consistent with the findings of Wen et al. (35), that patients enduring a longer period of pain preoperatively had a greater risk of developing kinesiophobia after surgery. The reason for this analysis may be that with the development of the accelerated recovery surgery concept (ERAS) and the advancement of pain management techniques, perioperative pain management in THA has achieved remarkable results, and the post-operative pain level of patients is generally at a relatively low and stable level, and the effect of the difference in the pain level on kinesiophobia may be weakened. In contrast, pain chronology indicates the duration of pain, reflecting patients’ long-term experience of pain and the long-lasting effects of chronic pain. Studies have shown (36, 37) that long-term chronic pain may lead to increased sensitivity of the central nervous system to pain signals, and that even mild post-operative pain may be perceived by the patient as severe pain, which induces fear and avoidance behaviors. In addition, patients with long years of pain may have developed negative expectations and catastrophizing thinking about pain and are more likely to develop false perceptions of the disease (38, 39). Therefore, the impact of long-term chronic pain may go beyond the pain itself, suggesting that healthcare professionals need to pay attention to the duration and degree of preoperative pain while focusing on the patients’ post-operative pain management, and to conduct more detailed psychological assessments and interventions for patients experiencing longer periods of preoperative pain.

#### 4.2.2 Anxiety

This study shows that patients with high anxiety levels are at a higher risk of developing kinesiophobia after surgery. This is due to the fact that higher levels of anxiety activate the sympathetic nervous system, causing increased heart rate and muscle tension, which exacerbate exercise avoidance behaviors, creating a vicious cycle of “anxiety-avoidance” (40). Patients usually have more negative expectations of pain and functional exercise, are more sensitive to pain and discomfort, and easily perceive functional exercise as a potential threat to aggravate pain, and misinterpret minor discomfort during rehabilitation as a worsening of the condition, leading to fear and avoidance of rehabilitation exercises (41). In addition, patients’ concerns about prognostic outcomes and the presence of post-operative complications can further exacerbate their anxiety. Therefore, in clinical practice, the assessment and management of anxiety can be incorporated into the prevention and intervention strategies of kinesiophobia to help patients effectively alleviate anxiety, reduce the risk of post-operative kinesiophobia, and improve patient prognosis.

#### 4.2.3 Rehabilitation self-efficacy

The results of this study showed that patients with higher rehabilitation self-efficacy scores were less likely to develop kinesiophobia. Rehabilitation self-efficacy, which refers to a patient’s specific confidence and beliefs about functional recovery after surgery, is one of the most important predictors of patient prognostic outcomes and an important influence on functional recovery in THA patients (42). Previous studies have shown (43) that self-efficacy is a key variable in predicting fearful avoidance behavior and plays a mediating role between an individual’s fearful emotions and avoidance behavior. Patients with low rehabilitation self-efficacy tend to lack confidence in various post-operative motor exercises and are more prone to feelings of helplessness and fear, and this negative psychological state will make them tend to avoid rehabilitation training, which in turn will affect the physical recovery process. In addition, a longitudinal study showed that rehabilitation self-efficacy can reduce patients’ fear of anticipated activities and better predict the long-term outcomes of post-operative THA patients (44). Therefore, improving rehabilitation self-efficacy is crucial for reducing the risk of kinesiophobia and improving prognosis, and healthcare professionals can help patients build confidence and improve self-efficacy by setting small achievable goals, providing positive feedback, and encouraging patients to share their successes.

#### 4.2.4 Active coping styles

The results of this study showed that a low active coping score was a risk factor for the development of kinesiophobia in THA patients after surgery. Coping styles refer to the cognitive and behavioral strategies that individuals adopt when facing stressful events (45). It has been shown (46) that positive coping styles are significantly and negatively associated with kinesiophobia. Patients who adopt positive coping styles usually have better psychological resilience and are better able to adapt to the challenges they face during the rehabilitation process, such as pain and joint discomfort. These patients are optimistic about the disease and the recovery process, and tend to actively seek support from the outside world and explore solutions to their problems. Before surgery, they are fully aware of the risks and expected outcomes, which reduces their uncertainty about the surgery and recovery process. In the post-operative period, they were able to actively participate in post-operative rehabilitation training, and their uneasiness and fear of rehabilitation exercises were relatively low. It has also been shown (47) that coping styles play a partial mediating role between self-efficacy and kinesiophobia, and that positive coping styles enhance patients’ self-efficacy and make them believe that they can safely perform rehabilitation exercises, which they will regard as an effective way to restore their health, rather than as a source of triggering fear. This positive perception and attitude will help patients face the rehabilitation process more comfortably and reduce the risk of kinesiophobia. Therefore, healthcare professionals should carefully observe patients’ emotional changes, provide them with timely psychological guidance and health education, guide them to adopt positive coping styles, encourage them to take the initiative to participate in rehabilitation training, improve their cognitive ability regarding the disease and exercise compliance, and provide them with support and assistance when necessary.

#### 4.2.5 Harris hip function

The results of this study show that patients with poorer hip function are more likely to develop kinesiophobia. The reason for this may be analyzed because the success of the surgery allowed patients who had been suffering from long-term pain due to hip disease to regain hip function and have their pain relieved. When patients do not suffer from significant pain during exercise, they are less likely to be fearful of exercise. In addition, the improvement of joint mobility and daily living ability will enhance patients’ self-confidence and self-efficacy, which will make them participate more actively in rehabilitation training and reduce their fear and avoidance behaviors toward exercise. On the contrary, patients with limited function may have a fear of exercise due to the psychological burden exacerbated by repeated frustration. In addition, patients with better hip joint function are usually able to complete rehabilitation training earlier and more smoothly, and earlier rehabilitation training also facilitates the recovery of post-operative joint function, forming a virtuous circle and thus effectively preventing the occurrence of kinesiophobia (48). This suggests that we can start rehabilitation training as early as possible under the premise of ensuring safety in the clinic and formulate an individualized rehabilitation plan to gradually improve the joint mobility and muscle strength of patients and promote functional recovery. At the same time, we should regularly assess the patients’ hip function as an important indicator for monitoring the rehabilitation process and predicting the risk of kinesiophobia, and timely adjust the rehabilitation program according to the changes in scores.

### 4.3 Scientificity and significance of constructing a nomogram prediction model for post-operative kinesiophobia in THA patients

In this study, five predictors were screened using Logistic regression (stepwise forward-backward method), and the nomogram prediction model was plotted to transform the complex regression equations into visualized graphs. The predictive model demonstrated excellent performance in terms of the evaluation metrics of the model. The area under the ROC curve of the prediction model was 0.947, the specificity was 0.873, the sensitivity was 0.911, and the Hosmer-Lemeshow test χ^2^ = 2.287, *P* = 0.971, and internal validation using the Bootstrap method yielded a ROC value of 0.912, which suggests that the prediction model has good post-operative kinesiophobia in THA patients with good predictive performance and accuracy. The calibration curves of both the model and internal validation showed consistency between predicted and actual values, and the DCA curves all indicated that the model had good clinical utility. Constructing a nomogram can help healthcare professionals to more intuitively understand the risk probability of a particular indicator occurring (49). By comprehensively assessing the impact of multiple factors on the risk of kinesiophobia, it is possible to more accurately predict which patients belong to the high-risk group, which is conducive to healthcare personnel taking targeted preventive and intervention measures according to the differences in different patient groups, to reduce the incidence of post-operative kinesiophobia, and to improve the long-term prognosis of patients.

In addition, as the predictors included in this model encompassed biological, psychological and behavioral dimensions, and the study subjects were all patients who underwent THA for the first time in the same hospital, the relative homogeneity of the study subjects reduced the interference of irrelevant variation on the results to a certain extent, resulting in the AUC values of the present study being higher than 0.9. However, despite the results of the internal validation supporting the good robustness of the model, in view of the limitations of the present limitations of study, the very high performance of the model still needs to be further validated by multicenter external studies in the future to ensure the general applicability and reliability of the model in a wider range of clinical settings.

### 4.4 Limitations and innovations of this study

Although the predictive model constructed in this study showed excellent performance, there are some limitations. Firstly, this study was a cross-sectional study, and all data were collected at the same post-operative time point; patients were not followed up. Secondly, the nomogram prediction model constructed in this study was not externally validated, and its external adaptability is not yet clear. Moreover, with the use of single-center convenience sampling, the representativeness of the sample may be affected by the level of diagnosis and treatment of a specific healthcare institution, the regional culture, and the characteristics of the patient group, which limits the generalizability of the results of the study to a certain degree and puts some constraints on its generalization and application. It is suggested that future studies may conduct longitudinal surveys, as well as enhance the external validity of the model through multi-center, random sampling research methods, and external validation to make the results more generalizable. In addition, this study used a biopsychosocial model to select the different dimensions of the study variables and visualized the complex predictive model in the form of a nomogram. This provides a convenient tool for individualized risk assessment in the clinic. In the future, the model can also be combined with the Accelerated Rehabilitation Surgical Theory (ERAS), which can be utilized to rapidly screen people at high risk for kinesiophobia, thus initiating multidisciplinary interventions at an early stage, and ultimately accelerating the patient’s recovery process.

## 5 Conclusion

The incidence of post-operative kinesiophobia in THA patients is high, and patients with a pain history duration of more than 1 year, poor hip function, low active coping, and poor rehabilitation self-efficacy are more likely to develop kinesiophobia in the post-operative period. The risk prediction model constructed in the present study has a good predictive efficacy, and it can serve as an effective tool for the early prediction of the risk of post-operative kinesiophobia in patients with THA in the clinical setting.

## Data Availability

The raw data supporting the conclusions of this article will be made available by the authors, without undue reservation.
